# A Novel Way for Synthesizing Phosphorus-Doped Zno Nanowires

**DOI:** 10.1007/s11671-010-9805-9

**Published:** 2010-09-28

**Authors:** Jingyun Gao, Qing Zhao, Yanghui Sun, Guo Li, Jingmin Zhang, Dapeng Yu

**Affiliations:** 1 State Key Laboratory for Mesoscopic Physics, and Electron Microscopy Laboratory, School of Physics, Peking University, 100871, Beijing, Peoples's Republic of China

**Keywords:** ZnO, Nanowires, P-doped, Zinc phosphate

## Abstract

We developed a novel approach to synthesize phosphorus (P)-doped ZnO nanowires by directly decomposing zinc phosphate powder. The samples were demonstrated to be P-doped ZnO nanowires by using scanning electron microscopy, high-resolution transmission electron microscopy, X-ray diffraction spectra, X-ray photoelectron spectroscopy, energy dispersive spectrum, Raman spectra and photoluminescence measurements. The chemical state of P was investigated by electron energy loss spectroscopy (EELS) analyses in individual ZnO nanowires. P was found to substitute at oxygen sites (P_O_), with the presence of anti-site P on Zn sites (P_Zn_). P-doped ZnO nanowires were high resistance and the related P-doping mechanism was discussed by combining EELS results with electrical measurements, structure characterization and photoluminescence measurements. Our method provides an efficient way of synthesizing P-doped ZnO nanowires and the results help to understand the P-doping mechanism.

## Introduction

As a kind of very potential candidate material for future short-wavelength optoelectronic devices, zinc oxide (ZnO) has stimulated intensive research interest in the past decades [1]. However, commercialization of ZnO remains in doubt due to great difficulties in achieving stable p-type-doped ZnO because of low doping efficiency [2] and instability of dopant in ZnO [3]. Phosphorus (P) has been proved to be one of the best dopant for p-type doping in ZnO; however, the doping mechanism remains controversial [4]. P has at least two stable configurations: complex (P_Zn_ + 2V_Zn_) formed by combination between antisite substitutional P (P_Zn_) and two zinc vacancies (V_Zn_); substitutional P at oxygen sites (P_O_) [4]. P_Zn_ + 2V_Zn_ were believed to be the effective configuration for p-type doping, which induces a shallow acceptor level in the band gap, while P_O_ was suggested to induce relatively deep acceptor level, not resulting in sufficient hole concentration in ZnO. Most of the p-type conductivity observed in P-doped ZnO is attributed to P_Zn_ + 2V_Zn_ until now [5,6] and high resistance of P-doped ZnO is always believed to be induced by P_O _[7]. However, experimental evidence for such assignment is rarely reported.

As building blocks for next generation nano-optoelectronic devices, p-type ZnO nanowires have attracted considerable attention recently [8,9]. P-doped p-type ZnO nanowires have been obtained by simple chemical vapor deposition method [10], direct carbothermal method [11], pulsed-laser deposition (PLD) [12], thermal evaporation [13], diffusion method [14] and hydrothermal method [15]. However, in all above reports, the configuration of incorporated P was not determined, leaving the doping mechanism unclear. Meanwhile, carbon (C), which was introduced more or less in most of the methods reported above, was demonstrated to be a big obstacle to realize p-type ZnO by forming n-type domains such as graphite clusters along the grain boundaries [16]. Thus, a kind of C-free method is highly needed to be more effective in p-type doping of ZnO.

In this work, a novel C-free approach was developed to synthesize P-doped ZnO nanowires. The configuration of incorporated P in ZnO was achieved from individual ZnO nanowires by electron energy loss spectroscopy (EELS) analyses. Possible P-doping mechanism was investigated by combining EELS results with electrical measurements.

## Experimental Section

ZnO nanowires were synthesized by directly decomposing zinc phosphate powder. Pure zinc phosphate powder (99%) was loaded in an alumina boat. The purity of zinc phosphate is rather low; however, as pointed out by the manufacturer, the main impurities in zinc phosphate are other compounds composed of phosphorus and zinc such as zinc pyrophosphate, as well as some lead, iron and other heavy metals. In addition, we checked the purity of zinc phosphate by EDS measurements, and no other elements can be detected except oxygen, zinc and phosphorus. We also checked the purity of the nanowires after growth by XPS, EDS and EELS measurements and we did not find other elements. As a result, we think that the impurity in the source does not affect the properties of nanowires severely. A piece of (001) sapphire slice was placed above the zinc phosphate powder as the collecting substrate. Then, the boat was placed at the center of a quartz tube and inserted into a rapid heating furnace. Argon was used to clean the furnace for 10 min and then set to 200 sccm as carrier gas during growth. The furnace was heated up to 1,050°C in 20 min and held for 1 min and then cooled down to room temperature naturally. Oxygen (2.6 sccm) was added as the reactive gas when the furnace temperature reached 1,050°C. After growth, the substrate was covered by a layer of wax-like product.

The samples were characterized by using scanning electron microscopy (SEM) (Quanta 200FEG), high-resolution transmission electron microscopy (HRTEM) (Tecnai F30) and energy dispersive spectrum (EDS). Raman spectra excited by a 514 nm laser (Renishaw inVia Raman Microscope Raman system) and photoluminescence (PL) using a He–Cd laser with a wavelength of 325 nm were measured. X-ray photoelectron spectroscopy (XPS) (AXIS-Ultra instrument, Kratos Analytical) analysis was carried out to investigate the chemical states of phosphorus in ZnO. EELS analyses were performed to determine the configuration of incorporated P in ZnO lattice.

## Results and Discussion

**Figure 1:**
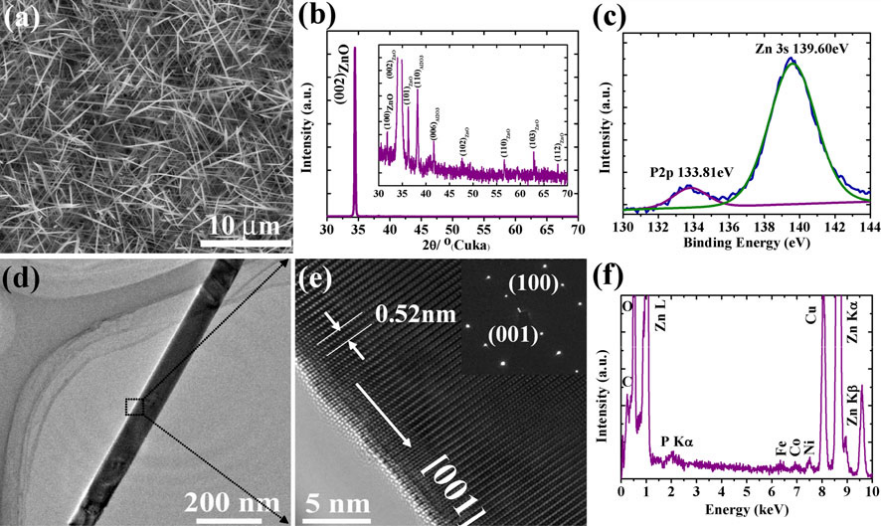
**a SEM image, b XRD spectrum and c XPS spectrum of P-doped ZnO nanowires**. The inset of **b** shows the detail of the peaks suppressed by (002) peak **d** Low-magnification TEM image and **e** HRTEM image of a typical P-doped ZnO nanowire. Inset of **e**: electron diffraction pattern of the nanowire **f** EDS spectrum of an individual P-doped ZnO nanowire.

Figure 1a shows a typical SEM image of the as-grown P-doped ZnO nanowires. The substrate is covered with nanowires with length up to several tens of micrometers and diameter in a range from tens to hundreds nanometers. All peaks in the X-ray diffraction (XRD) spectrum of the as-grown samples (Figure 1b) correspond to ZnO lattice, and no additional peak related to secondary phase appears, indicating purity of our sample. XPS results (Figure 1c) show P peak at 133.56 eV, proving preliminarily the incorporation of P into the as-grown nanowires.

Low-magnification TEM image of a typical P-doped nanowire is shown in Figure 1d. The P-doped nanowires have a uniform diameter about 100 nm from bottom to top and a smooth surface with no cluster attached on it. High-resolution TEM image (Figure 1e) shows that the as-grown P-doped ZnO nanowires are single crystal without amorphous layer on the surface. The lattice spacing along the growth direction is 0.52 nm, corresponding to the planar spacing between two (001) planes of ZnO, which indicates that the as-grown samples are ZnO nanowires rather than Zn_3_P_2_ or other phases. As shown in the electron diffraction pattern in the inset of Figure 1e, the P-doped ZnO nanowires grow along [001] axis. No second phase or cluster could be detected in the electron diffraction pattern. Zn and O peaks dominate the EDS spectrum from a single P-doped ZnO nanowire (Figure 1f) with P signal, indicating the existence of P in individual nanowires. The peak of C and Cu comes from copper grid and Fe and Co and Ni are from Tecnai F30. By analyzing the EDS spectrum, the content of phosphorus in the nanowire was quantified as P: Zn = 1.4% (Atomic Ratio), corresponding to the P concentration of ~10^20^ cm^-3^.

**Figure 2:**
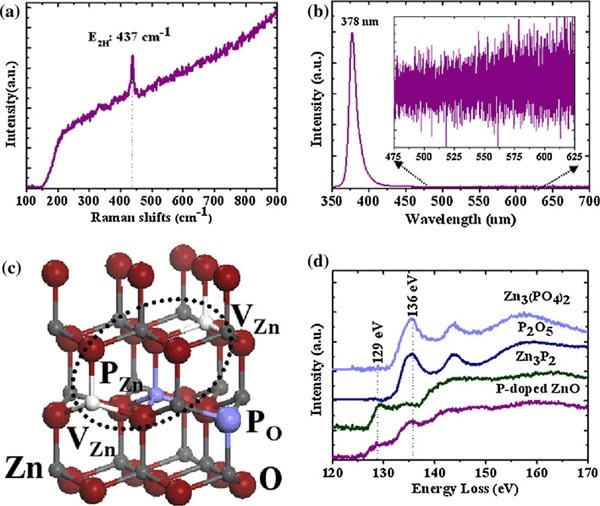
**a Raman spectra of P-doped ZnO nanowires b PL spectra of P-doped ZnO nanowires at room temperature**. Inset: detail of the defect-related emission part in PL **c** Atomic configurations of P_O_ and P_Zn_ + 2V_Zn_ in ZnO, respectively. *Big red balls* represent oxygen atoms and *small gray balls* represent zinc atoms. Phosphorus atoms and Zinc vacancy are marked by P and V_Zn_, respectively **d** EELS spectra obtained at the P L_23_ peak in P-doped ZnO nanowires with the referenced P L_23_ peaks in Zn_3_(PO_4_)_2_, P_2_O_5_ and Zn_3_P_2_, respectively.

Raman and PL measurements were carried out to characterize the quality of the as-grown P-doped ZnO nanowires. There are two common peaks in ZnO Raman spectra: one at 437 cm^-1^ corresponding to E_2H_ mode and one broad peak at 580 cm^-1^, which is a combination of E_1LO_ and A_1LO_ modes and usually been found to be enhanced by disorder [17]. As shown in Figure 2a, only the peak at 437 cm^-1^ appears in the Raman spectra of P-doped ZnO nanowires. The absence of 580 cm^-1^ peak is a signal of good ZnO lattice quality grown by this method.

In Figure 2b, the PL spectrum of P-doped ZnO nanowires at room temperature has only one peak centered at 380 nm, which is the near band edge (NBE) emission due to recombination of free excitons (FX). No defect-related emission is observed, especially the commonly observed peak at 520 nm caused by oxygen vacancy and the peak around 480 nm caused by zinc vacancy [18]. Magnified spectrum from 475~625 nm part (inset of Figure 2b) demonstrates that the defect-related emission disappears. The absence of such defect-related emission suggests the good quality of the as-synthesized P-doped ZnO nanowires.

Hereto, successful synthesis of P-doped ZnO nanowires by decomposing zinc phosphate powder was demonstrated. Next, we investigated the configuration and chemical state of incorporated P in the as-grown P-doped ZnO nanowires by EELS analyses, which can give information of chemical valence and neighboring environment. Atomic configurations of P_O_ and P_Zn_ + 2V_Zn_ in ZnO are shown in Figure 2c. P L_23_ peak appears in the EELS spectra (Tecnai F30 together with a Gatan imaging filter) of P-doped ZnO nanowires (Figure 2d), which is a evidence for the incorporation of P in ZnO nanowires. P L_23_ peak in Zn_3_P_2_, P_2_O_5_ and Zn_3_(PO_4_)_2_ was also measured for comparison and shown in Figure 2d as well, where P adopts chemical valence -3, +5 and +5, respectively. For reference samples, Zn_3_P_2_ nanowires, P_2_O_5_ powder and Zn_3_ (PO_4_)_2_ powder were used, respectively. From Figure 2d, it can be seen that the P L_23_ peak from P_2_O_5_ is almost the same as those from Zn_3_(PO_4_)_2_, because of the same chemical valence of P. The peak around 136 eV is demonstrated to be associated with +5 state of P [19]. In Figure 2d, the one mostly similar to the P L_23_ peak in P-doped ZnO nanowires is those from Zn_3_P_2_, with a little difference on the intensity of the peak around 129 eV. The peak at 129 eV is attributable to the -3 state of P [19]. The appearance of the peak at 129 eV and the agreement between the P L_23_ peak from P-doped ZnO nanowires and Zn_3_P_2_ demonstrate that a part of P in P-doped ZnO nanowires adopts chemical valences of -3 as in Zn_3_P_2 _[19], corresponding to substitutional P at an O site, forming Zn–P bonds with surrounding Zn atoms. The intensity difference between P L_23_ peaks from P-doped ZnO nanowires and Zn_3_P_2_ results from the difference between the fine configurations of P in these two cases. In addition, the peak at 136 eV related to +5 state of P also appeared in EELS spectrum of P L_23_ peak from P-doped ZnO nanowires, indicating the presence of antisite P with chemical valence of +5. The role of PO and P_Zn_ will be discussed after the electrical measurements section.

EELS has been wildly used to investigate the chemical environment of magnetic dopant in ZnO [20,21], while there is no report of EELS analyses on p-type dopant mainly due to low doping concentration. As an alternative, X-ray photoelectron spectroscopy (XPS) was always used to characterize the chemical state of dopant. However, as we know, XPS reflects the mixed information from the entire substrate, which cannot be corresponded to state of dopant in individual nanowire. Compared to XPS, EELS analyses on individual nanowire provide unambiguous information about the chemical state of dopant in single nanowire and are effective to understand the doping mechanism related to such state.

**Figure 3:**
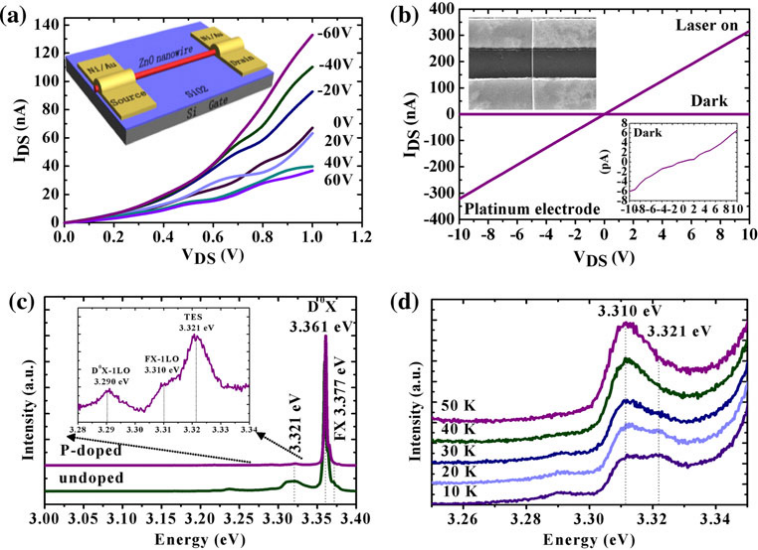
**a I_DS_–V_DS_ plots of P-doped ZnO nanowire FETs measured under UV illumination**. Inset: The schematic illustration of the measured device **b** I_DS_–V_DS_ plot of the P-doped ZnO nanowire FETs transistor with platinum electrodes. *Lower right* inset: Enlarged I_DS_–V_DS_*curve in dark*. *Upper left* inset: SEM image of a typical device **c** NBE of pure and P-doped ZnO nanowires at 10 K. Inset: enlarged part of the peaks around 3.31 eV for P-doped ZnO nanowires **d** Temperature-dependent PL spectra of P-doped ZnO nanowires with the evolution of two separate peaks around 3.31 eV.

To investigate the doping mechanism of P incorporated into the P-doped ZnO nanowires, back-gate field-effect transistors (FETs) measurements were conducted (inset of Figure 3a). Silicon (n±Si) substrates covered by a 300 nm SiO_2_ layer served as the back-gate and dielectric gate oxide, respectively. Single P-doped ZnO nanowire was dispersed on the substrates. A layer of 20 nm Ni and 80 nm Au was deposited on the two ends of the nanowire as the contact by e-beam lithography and sputtering. The upper left inset of Figure 3b shows a typical image of a single P-doped ZnO nanowire FET. We fabricated twenty FETs in all and found that the as-grown P-doped ZnO nanowires are high resistance. The resistance was so large that the current between drain and source (I_DS_) could not be modulated by gate voltage (V_G_), and we could not make sure whether the high resistance came from the nanowire itself. To rule out the other factors that may contribute to the high resistance observed in the P-doped nanowires, we measured the device under UV illumination. I_DS_ as a function of voltage between drain and source (V_DS_) under different gate voltages (V_G_) was plotted in Figure 3a, where the gate voltages modulated the current through the nanowire obviously. Thus, we made sure that there was no problem with our measurement process. The presence of the intersection between I_DS_ at V_G_ = 0 V and V_G_ = 20 V is due to the weak modulation of small V_G_ on weak I_DS_. To further rule out the effect of contact resistance on the measurements, we fabricated platinum electrode at two ends of ZnO nanowire by focused ion beam (FIB)-induced deposition technique, which have previously been proven to be ohmic contact with ZnO [22]. Then, we measured the device in dark and under UV illumination (see Figure 3b), respectively. We can see that the I_DS_ is really weak in dark and the resistance is estimated to be ~10^4^ Ωcm. However, the nanowire forms ohmic contact with the platinum electrode and the contact resistance is not the cause of the high resistance observed above. The I_DS_ is not perfect linear due to low carrier concentration of as-grown nanowire in dark. As concluded from the above discussions, we believe that the nanowires are high resistive. However, we cannot determine the conductive type of our P-doped ZnO nanowires in dark according to the measurements under UV illumination due to the effect of photo-generated carriers [23].

Combining electrical measurements with EELS results and structure characterization, the doping mechanism of P is discussed as follows. From Raman and PL characterization, P-doped ZnO nanowires are of high quality. The possibility of imperfect lattice inducing high resistance of P-doped ZnO nanowires can be excluded. The native defects in P-doped ZnO nanowires are limited, thus the compensation effects from native defects are suppressed. Thus, P did not generated sufficient hole in as-grown P-doped ZnO nanowires though it mainly substitutes at oxygen sites from EELS analyses, which mostly because it induces a deep acceptor level in the ZnO band and may not provide effective conductive hole to contribute to p-type doping. In addition, due to the lack of zinc vacancy, the P_Zn_ cannot form effective P-doping configuration of P_Zn_ + 2V_Zn_ and does also not generate sufficient hole.

To confirm the effects of P-doping on electronic structure of ZnO, the PL measurements at 10 K were performed. The PL spectrum of P-doped ZnO nanowires at 10 K is shown in Figure 3c with the enlarged detail for the peaks around 3.310 eV shown in inset. The PL spectrum of pure ZnO nanowires at 10 K is also shown for reference. The free-exciton (FX) peak locates at 3.377 eV with its first longitudinal-optical (LO) phonon replicas at 3.310 eV, consistent with other reports [24]. The difference between FX and FX-1LO is only 67 meV, smaller than the LO-phonon energy of ZnO, due to energy softening of 1LO-phonon [25]. The neutral–donor-bound exciton (D_0_X) centered at 3.361 eV dominates the near band edge emission (NBE) of P-doped ZnO nanowires with D_0_X-1LO at 3.290 eV. Neutral-acceptor-bound peaks around 3.35 eV, which were always found in P-doped ZnO [26,27], were not observed here. The peak at 3.321 eV is always observed in P-doped ZnO films and attributed to free electron to acceptor (FA) transitions [26,27]. To investigate the origin of 3.321 eV peak, the temperature-dependent PL measurements were performed. In Figure 3d, as the temperature rising from 10 to 50 K, the peak at 3.321 eV decreased rapidly and disappeared at 40 K with only FX-1LO peak left. It was reported that FA emission is often accompanied with DAP (donor–acceptor pair) emission in ZnO at low temperature [24]. The DAP emission can transform into FA with increasing temperature due to the smaller donor binding energy than acceptor energy. Thus, FA intensity was relatively enhanced compared to D_0_X and TES as increasing temperature, though the absolute intensity of whole spectrum decreased. The FA peak will remain in PL spectrum until the acceptors are thermally ionized at temperatures above 200 K. Here, the peak at 3.321 eV decreased rapidly as the increasing temperature and disappeared at 40 K, which was not the characteristic of FA. As a result, the peak at 3.321 eV could not be assigned to FA. In addition, the peak at 3.321 eV also appeared in PL of pure ZnO nanowires as shown in Figure 3c, which could be assigned to two-electron satellite (TES) emission, which often appeared in low-temperature PL of ZnO and disappeared at the temperature around 50 K [28]. The absence of P-related peak in PL of P-doped ZnO nanowires may confirm our discussion about P-doping mechanism in above section: P_O_ induces a deep acceptor level in the ZnO band and may not provide effective conductive hole to contribute to p-type doping. P_Zn_ cannot form effective P-doping configuration of P_Zn_ + 2V_Zn_ due to the lack of zinc vacancy. To realize p-type doping in P-doped ZnO, appropriate growth conditions must be adopted to form P_Zn_ + 2V_Zn_, which is the effective configuration of P for the p-type doping. Our results confirm the theoretical results related to P-doping mechanism in ZnO [4].

As we introduced, we developed a kind of C-free method to synthesize P-doped ZnO nanowires. The detailed characterization on the effect of C was not performed because C presented in most of characterization method. C is usually used as the standard reference in XPS measurement and a thin C layer covers on the copper grid used in EELS analyses performed in TEM. However, we believe that the absence of C in the growth process would be very effective to avoid the possible effect induced by C [16]. Such kind of C-free method can be applied to synthesize other materials nanowires by decomposing-related compound.

## Conclusions

In summary, P-doped ZnO nanowires were successfully synthesized by a C-free method via directly decomposing zinc phosphate powder. As-grown samples were single-crystalline ZnO nanowires grown along [001] direction. Phosphorus incorporated into individual ZnO nanowires, which were directly confirmed by EELS analyses. EELS analyses demonstrated that P incorporated substitutionally at O sites in the as-grown P-doped ZnO nanowires with the presence of P_Zn_. As-grown P-doped ZnO nanowires showed high resistance, which because that P_O_ induced a deep acceptor level in the band gap and P_Zn_ cannot form effective P-doping configuration of P_Zn_ + 2V_Zn_ due to the lack of zinc vacancy. Our method provides a novel approach to synthesize P-doped nanowires, and the investigation results help to understand the p-type-doping mechanism of ZnO nanowires.
